# Preclinical development of tumor-infiltrating lymphocyte therapy for ovarian cancer

**DOI:** 10.1186/2051-1426-3-S2-P48

**Published:** 2015-11-04

**Authors:** Donastas Sakellariou-Thompson, Cara Haymaker, Marie-Andree Forget, Amir Jazaeri, Patrick Hwu, Chantale Bernatchez

**Affiliations:** 1 The Department of Melanoma Medical Oncology, The University of Texas MD Anderson Cancer Center, Houston TX, USA; 2 The Department of Gynecologic Oncology and Reproductive Medicine, The University of Texas MD Anderson Cancer Center, Houston TX, USA

## Background

Immunotherapy has become an effective cancer therapy, particularly in the case of checkpoint blockade and adoptive T cell therapy (ACT). ACT exploits the presence of tumor-infiltrating lymphocytes (TIL) by exponentially expanding their numbers *ex vivo* and re-infusing them into the patient in an autologous setting. With the effectiveness of TIL therapy already well established in multiple Phase II studies in melanoma, there is a push to translate it to other malignancies such as ovarian cancer (OvCa) [1].

## Methods

The presence of TIL is correlated with greatly increased survival in OvCa [2,3] suggesting that TIL effectively control the disease and provide a rationale to test TIL therapy in this setting. To assess the feasibility, we characterized the immune component of OvCa, explored the ability to grow & expand TIL from tumor fragments, and tested their functionality.

## Results

Extensive flow cytometry analysis detected a robust, activated T cell infiltrate that can be grown from OvCa samples obtained pre- and post-chemotherapy. The addition of an agonistic anti-41BB antibody to the cultures preferentially increased CD8^+^ TIL outgrowth as well as favored the expansion of NK cells. Importantly, success rate of TIL growth was increased from 40% to 90% for cultures grown without and with anti-41BB respectively. It was established next that the CD3^+^ TIL initially grown with anti-41BB could be rapidly expanded at least 1000 fold over two weeks. Finally, the rapidly expanded T cells exhibited anti-tumor capabilities in the context of re-directed killing assays.

## Conclusions

In conclusion, further flow cytometry analysis to identify other agonistic and inhibitory targets is needed along with additional *in vitro* and *in vivo* experiments. However, the initial data suggest that TIL therapy for OvCa could be a viable therapeutic option in the future.
